# Stress and the Level of Fear of Childbirth Among Pregnant Women in Poland During the Pandemic—The Importance of a Sense of Coherence and Partner Support

**DOI:** 10.3390/jcm14248628

**Published:** 2025-12-05

**Authors:** Joanna Dymecka, Dagmara Pawłowska, Anna Machnik-Czerwik, Radosław Dziedzic

**Affiliations:** 1 Department of Health Psychology and Quality of Life, Institute of Psychology, Opole University, Staszica Square 1, 45-015 Opole, Poland; jdymecka@uni.opole.pl; 2 Student Opole University, 45-015 Opole, Poland; bremains@gmail.com; 3 Department of General Psychology and Human Development, Institute of Psychology, Opole University, 45-015 Opole, Poland; radoslaw.boczon@uni.opole.pl

**Keywords:** fear of childbirth, sense of coherence, social support, pandemic stress

## Abstract

**Background/Objectives:** Stress experienced by pregnant women during the pandemic may be associated with an increased level of fear of childbirth. Psycho-social resources such as a sense of coherence and social support may be associated with better psychological outcomes. The aim of the study was to examine whether there is a relationship between pandemic stress, fear of childbirth, sense of coherence, and social support in pregnant women and whether pandemic stress, sense of coherence, and social support were explanatory variables for fear of childbirth. **Method:** The study involved 232 pregnant women. The following research methods were used in the study: the Berlin Social Support Scales (BSSS), Sense of Coherence Scale (SOC-29), Labor Anxiety Questionnaire (KLP II), and Pandemic-Related Pregnancy Stress Scale (PREPS). **Results:** The study showed that pandemic stress is positively correlated with infection and Preparedness Stress (infection stress: r = 0.57 ***; Preparedness Stress: r = 0.34 ***). Stress and fear of childbirth are negatively related (infection stress: r = 0.33 ***; Preparedness Stress: r = 0.4 ***), and fear of childbirth is negatively associated with the sense of coherence (r = −0.33 ***) and, to a small extent, with some types of social support. The research shows that obstetric variables related to the organization of childbirth are of significant importance for the psychological functioning of women. Preparedness Stress and sense of coherence were explanatory variables for fear of childbirth in multiple linear regression analyses (F(5, 224) = 19.48; adjusted R^2^ = 0.29). **Conclusions:** The sense of coherence turned out to be a more important resource for the psychological functioning of pregnant women than social support. The lack of control over such an important event as childbirth is associated with higher stress and the inability to use resources. In clinical practice, it is important to strengthen the resources for women who experience a strong fear of childbirth.

## 1. Introduction

Pregnancy is a time of many changes in a woman’s life, both physical and mental. Pregnancy, especially the first one, is a time of very intense emotional experiences. The emotional functioning of pregnant women depends on many factors. Pregnancy itself, even if the woman does not have any major problems or complications, can be a source of increased stress and anxiety [1,2]. Studies indicate that during pregnancy, women are primarily concerned about the possibility of miscarriage, followed by childbirth, going to the hospital, or the health of the unborn child [3]. One of the factors leading to increased stress in pregnant women was the COVID-19 pandemic, which began in early 2020 and became the largest public health crisis in the 21st century.

The COVID-19 pandemic has led to an overload of healthcare systems in most countries and to the emergence of mental health problems for many people around the world. During the pandemic, people feared infection, death, transmission of the virus to loved ones, and quarantine and isolation [4]. The pandemic, as a difficult situation, contributed to severe emotional stress [5,6]. Apart from that, pre-pandemic data indicate that pregnant women experience greater stress compared to the general population [7]. Our another study [8] showed that the level of stress in pregnant women, measured using the PSS-10 scale, is higher compared to the results obtained from pregnant women before the pandemic [7]. It is also higher than that of the average for the Polish population [9]. Studies show that pregnant women experience moderate-to-high levels of stress during the COVID-19 pandemic [10,11,12]. This is probably the result of the interaction of social, economic, and health factors [6].

The COVID-19 pandemic has caused stress due to the very fact of the spread of the virus, but also due to the restrictions to limit its transmission [4]. Restrictions and isolation have adversely affected the functioning of the community, including vulnerable groups such as pregnant women, and have exacerbated stress symptoms [10]. Women were worried about their baby, about the isolation during childbirth, and about the loss of social networks in the postpartum period [13]. In addition, pregnant women experienced significant changes in pregnancy management, labor, and postpartum care. Many healthcare facilities limited in-person visits and introduced teleconsultations [14]. The threat of infection and changes in healthcare were a significant burden for women [8]. Studies have shown that the sense of security of pregnant women and those planning a pregnancy was significantly lower than before the pandemic. The alarmingly low birth rate during the pandemic—the lowest since World War II—may be evidence of how widespread these feelings were [15].

During the COVID-19 pandemic, two main sources of stress were demonstrated in pregnant women: stress related to the risk of infection and stress related to the feeling of being unprepared for childbirth due to pandemic-related restrictions [6,8,16,17]. Studies show that about one third of pregnant women experienced stress during the COVID-19 pandemic, both related to changes in the organization of labor and the risk of infection. In addition to confirming factors that were previously considered to contribute to prenatal stress, risk factors specific to the pandemic were also identified [6,8]. One of the reasons for the higher level of stress was the limitation of family births due to epidemiological reasons and problems with the implementation of the birth plan. During the COVID-19 pandemic, due to hospital restrictions, women giving birth experienced higher levels of stress and fear because they had to cope alone [14], and visits from loved ones were suspended in hospitals. The literature has shown that the support of the partner, mother, other family members and friends of the pregnant woman in the perinatal period is important for reducing stress and anxiety [18]. However, giving birth alone, without the support of a partner, is associated with higher levels of stress and higher pain and may even end in a cesarean section [19]. All of this may simultaneously lead to a higher level of fear of childbirth.

Despite the fact that perinatal care in developed countries is at a high level, fear of childbirth is a common problem affecting the health and well-being of women before and during pregnancy, as well as after childbirth [20]. Pregnant women’s fears related to childbirth may concern the health and life of the baby, pain, and injuries, including damage to the perineum, but also giving birth to a child with defects or inappropriate behavior of medical personnel. Another study showed that fear related to childbirth was related to a lack of trust in the staff (73%), fear of one’s own incompetence (65%), fear of death of the mother, newborn, or both (55%), severe pain (44%) or loss of control over oneself (43%). In the case of women who have already given birth, fear was most often related to negative experiences from the previous childbirth, e.g., pain, complications during pregnancy or childbirth, having a disabled child, or an emergency cesarean section [21].

In addition, severe stress and anxiety during pregnancy can affect the development of the relationship between the mother and the child, as well as the relationship between the pregnant woman and her closest family, especially her partner. The mother’s emotional stress can also increase the risk of emotional problems in the child [21,22]. Therefore, it is important to identify factors that protect women from severe fear of childbirth.

Childbirth can be a particularly stressful event that causes fear and anxiety in women with low stress resistance. According to the concept of Lazarus and Folkman [23], personal stress resistance affects the perception of threat by an individual and coping with it. On the other hand, according to the concept of Antonovsky [24], there are factors that determine an individual’s resistance to stress and difficult situations, and one such factor is the sense of coherence. The sense of coherence is a theoretical construct introduced by Aaron Antonovsky, which means a person’s “global orientation that expresses the extent to which one has a pervasive, enduring though dynamic feeling of confidence that (comprehensibility) the stimuli deriving from one’s internal and external environments in the course of living are structured, predictable and explicable; (manageability) the resources are available to one to meet the demands posed by these stimuli; and (meaningfulness) these demands are challenges, worthy of investment and engagement” [24]. According to Antonovsky, an individual with a high level of sense of coherence perceives stressors as challenges and is able to effectively use resources in response to anticipated difficulties in order to modify their actions and thus maintain health despite emerging problems. A high level of SOC strengthens a person’s resilience, quality of life, and well-being [25]. According to Antonovsky’s concept, the sense of coherence is a relatively constant feature and does not change significantly throughout an individual’s life.

To date, many studies have been conducted analyzing the relationship between the sense of coherence and health and well-being. Their results indicate that a high level of SOC is associated with perceived good health, especially mental health. Most researchers claim that the sense of coherence is a significant predictor of mental health and well-being [26]. A systematic review of studies using the SOC questionnaire from 1992 to 2003 showed that a high level of sense of coherence protects against anxiety, depression, burnout, and hopelessness [27]. Empirical data confirm that the sense of coherence is a source of individual resilience. It affects life satisfaction, acceptance of unavoidable difficulties, and control over the situation in which the individual finds themselves. SOC can act as a protective factor against the harmful effects of perceived stress [28,29]. The sense of coherence is important in all stressful situations, but its effect is most important in people under severe stress. A strong SOC is especially important when the individual experiences very difficult situations [30,31].

As studies indicate, the sense of coherence is also an important resource for women during pregnancy, in the perinatal period and after childbirth. Kroemke and Mateusiak [32] showed that women who had a higher sense of coherence experienced fewer depressive symptoms, both during pregnancy and after childbirth, in the postpartum period. It has also been shown that the sense of coherence may also be related to the course of labor. Women with a high sense of coherence more often decide on natural childbirth and less often ask for anesthesia during labor [33]. According to Ferguson and colleagues [34], the fact that a woman is characterized by a high SOC reduces the likelihood of a cesarean delivery by half. Women with a low SOC, on the other hand, have a more negative attitude towards childbirth, focus their attention on the childbirth itself, and often worry [35]. Another study also showed that women with a higher sense of coherence decided to give birth naturally more often [36], and they experienced complications during labor less often [37]. All these studies suggest that SOC is an important resource for pregnant women. In the perinatal period, the sense of coherence can be considered a variable that explains differences in fear of childbirth.

Social support is also a significant factor for the health and psychological comfort of pregnant women. It is an important relational resource that is important at every stage of a person’s life. It can be an important factor in determining proper development and psychological functioning. Social support can be defined as a type of help that is provided to individuals or groups. The aim of support is to mobilize the strength of individuals and groups, their potential, and their resources so that they are able to cope with problems on their own. Support is also sometimes defined as a certain social interaction that is undertaken by participants in a stressful or problematic situation [38,39].

In the literature, four main types of social support are distinguished: instrumental, material, informational, and emotional support. Instrumental support is said to occur when an individual receives help in solving a specific problem. Material support is a situation in which a given person offers shelter, food, clothing, and money to the supported person. Informational support is associated with obtaining information or guidance. Emotional support, on the other hand, is associated with providing comfort and showing feelings to another person [40]. The role of received support and perceived support is also analyzed. The most important element in the case of social support is the subjective feeling of being loved and important to others [39].

Pregnant women can receive support from close people, healthcare workers, or other institutions, but support from a partner seems to be particularly important. Women who have the support of a partner perceive childbirth experiences as positive [41]. Social support from a partner can significantly reduce the fear of childbirth and is associated with life satisfaction [41,42]. A lack of support from partner can contribute to postpartum depression and may reduce the quality of life [43]. Moreover, studies have shown that women who had a planned cesarean section received a higher level of social support than women who gave birth naturally [41]. In the case of women who gave birth for the first time, the level of support provided was also higher [44]. Support can also have a beneficial effect on the mother–child relationship [45]. It has also been shown that partner support is one of the most important protective factors in women with high-risk pregnancies [46]. In addition, the help and presence of a partner can make a woman feel beautiful and accepted. This in turn brings many positive effects, such as longer breastfeeding or greater ease in enduring various inconveniences of pregnancy and the pain and hardship of childbirth [46,47].

Research conducted during the COVID-19 pandemic has shown that pregnant women experienced higher levels of stress and increased fear of childbirth [8,48,49]. Personal and psychosocial resources play a significant role in coping with stress during the COVID-19 pandemic. Previous studies have demonstrated the importance of factors such as hardiness, self-efficacy, and self-esteem in the mental health of pregnant women. It has been shown that the sense of coherence played a significant role in coping with pandemic-related stress and fear of COVID-19 in other groups [50].

Although several studies have examined stress and fear of childbirth during pregnancy, research focusing on the interplay between pandemic-related stress, the sense of coherence, and social support in predicting fear of childbirth remains limited. Previous work by Dymecka et al. [49] demonstrated that pregnant women in Poland experienced elevated stress levels and that psychosocial resources such as hardiness help with coping. However, these studies did not specifically examine how the sense of coherence and different sources of social support, including partner support, interact to influence fear of childbirth. It is also worth noting that examining social support is particularly important during pandemics or other public health crises, as access to support may be restricted—for instance, due to limitations on family presence during childbirth or reduced opportunities for social interaction. Addressing this gap, the current study investigates these relationships in a systematic way, aiming to examine key psychological and relational variables related to fear of childbirth. By doing so, the study contributes to the academic understanding of prenatal stress and fear, and provides a basis for clinical strategies to support pregnant women. The aim of our research was to determine the relationship between the analyzed variables and to determine the predictors of fear of childbirth.

Hypothesis:

**H1.**  *Perceived stress will be positively associated with fear of childbirth.*

**H2.**  *Sense of coherence will be negatively associated with fear of childbirth and with perceived stress.*

**H3.**  *Partner support will be negatively associated with fear of childbirth and with perceived stress.*

**H4.**  *Psychosocial resources (sense of coherence and partner support) together with perceived stress will jointly account for the variability in fear of childbirth.*

**H5.**  *Selected obstetric variables (e.g., parity, pregnancy complications) and sociodemographic variables (e.g., education, age) will be significantly associated with fear of childbirth.*

## 2. Materials and Methods

### 2.1. Subjects and Study Procedure

The study cohort consisted of 232 pregnant women. The study was conducted between 11 March 2022 and 1 March 2023. Due to the epidemiological threat, every effort was made to ensure that the study was completely safe for participants. Therefore, it was decided to recruit respondents via the Internet, by a Google Form. Two methods of recruitment for research were used: study assistants were asked to share the survey on social media platforms for pregnant women, and the snowball method was used. The study participants were informed that the study was completely anonymous and voluntary, and all respondents provided informed consent before completing the questionnaire.

The inclusion criteria were being pregnant at the time of the study, being at least 18 years old, and giving informed consent to participate. Exclusion criteria included not completing the questionnaire or reporting a diagnosis of a severe psychiatric disorder. Power analysis indicated that the sample size (N = 232) was sufficient to detect medium effect sizes with approximately 80% statistical power in correlational analyses (r ≥ 0.30), multiple regression analyses including two predictors (f^2^ = 0.15), and independent-samples *t*-tests comparing two groups of a similar size (Cohen’s d = 0.5).

To minimize missing responses, all items in the online questionnaire were made mandatory for submission. In cases where responses were missing, a central value approach was used. Participants who skipped more than two items were excluded from subsequent analyses. For participants who omitted fewer than three responses, missing data were imputed using the median values of the respective response scales. The survey was configured to permit only one response per participant, thereby preventing duplicate entries and ensuring data integrity.

The study received the consent of the Ethical Committee of the University of Opole (KEBN 15/2021, approved on 26 May 2021) and was conducted in accordance with institutional ethics standards and the Declaration of Helsinki.

### 2.2. Research Tools

The following research tools were used in the study:

An author’s survey on sociodemographic and obstetric variables was used. It consists of 22 questions and includes demographic and obstetric variables—age, education, number of pregnancies and deliveries, week of pregnancy, and chronic diseases—and variables related to the pandemic—fear of COVID-19 infection or possible difficulties related to the epidemiological situation (e.g., inability to have a family birth).

The Pandemic-Related Pregnancy Stress Scale (PREPS), by Preis, Mahaffey, and Lobel [47,51], adapted in Polish by Ilska et al. [16], was used to examine prenatal stress during the COVID-19 pandemic. The subjects responded to the questions using a five-point Likert scale, where 1 means “very little” and 5 means “very much”; the higher the result, the higher the level of stress. The questionnaire consists of 15 statements and three subscales: Perinatal Infection Stress I-S (5 items, α = 0.874, ω = 0.878), Preparedness Stress P-S (7 items, α = 0.855, ω = 0.878), and Positive Appraisal POS (3 items, α = 0.780, ω = 0.793). The reliability of the PREPS Questionnaire is satisfactory for the whole scale, at α = 0.915 and ω = 0.917, and for the stress model, (I-S + P-S) α = 0.908 and ω = 0.910.

The Labor Anxiety Questionnaire (KLP II) was used to examine the fear of childbirth in pregnant women. It was created by Putyński and Paciorek [52]. The questionnaire consists of 9 statements. The examined person responds to each of them on a 4-point scale: from definitely yes to definitely no. The result of the examined person ranges from 0 to 27 points, the higher the result, the higher the level of fear of childbirth. The reliability index is satisfactory (α = 0.811, ω = 0.815).

The Sense of Coherence Scale (SOC-29) by Antonovsky [53] is used to examine the sense of coherence. It consists of 29 questions. After reading a given statement, the subjects mark the answer on a seven-point scale with described extremes; the higher the result, the higher the level of sense of coherence. The questionnaire consists of three subscales concerning the sense of comprehensibility (α = 0.750, ω = 0.759), manageability (α = 0.784, ω = 0.793), and meaningfulness (α = 0.801, ω = 0.816). The Polish adaptation was created by Koniarek, Dudek, and Makowska [54]. Cronbach’s alpha coefficient for the whole scale (α = 0.897, ω = 0.902) indicates that the reliability of the questionnaire is satisfactory. It is also characterized by high validity.

The Berlin Social Support Scales (BSSS) by Schwarzer and Schutz [55], in the Polish adaptation by Łuszczyńska et al. [56], were used to examine social support. The BSSS consists of 6 different scales that can be used both together and separately. The available scales are Perceived Available Support, Need for Support, Support Seeking, Actually Received Support (Recipient), Provided Support (Provider), and the Protective Buffering Scale—Support Provider/Support Recipient. In this study, Actually Received Support was used. The scale contains 15 questions divided into emotional (9 items, α = 0.919, ω = 0.928), instrumental (3 items, α = 0.837, ω = 0.842), and informational social support (2 items, α = 0.864, ω = 0.868), and satisfaction with social support received contained a single item. Each of them can be answered on a four-point scale, where 1 means that the statement is completely untrue, and 4 means it is completely true; the higher the result, the higher the level of social support. The reliability of the whole tool (α = 0.954, ω = 0.959) is also satisfactory.

### 2.3. Statistical Analysis

Statistical analyses were performed in Statistica software, version 13, and JAMOVI version 2.3 [57], with use of certain R [58] packages, psych [59], car [60] and ggcorrplot [61], used in R Studio [62]. Missing data were filled in using a central value for the answering scale, only for those participants who had a maximum of two missing answers to any question coming from psychological questionnaires. First, descriptive statistics were studied. The minimum and maximum, mean, skewness, kurtosis, and standard deviation were calculated. The Shapiro–Wilk test was performed to examine the compliance of the distribution with the normal distribution. Parametric tests were used to analyze the results. Pearson r correlations with Bonferroni correction were used to examine the relationships between variables. Student’s *t* test for independent samples was used to check the differences between groups. Regression analysis was performed to check whether stress related to infection, stress related to childbirth, positive reappraisal, sense of coherence, and social support from the partner are explanatory variables of fear of childbirth. For power analysis, we used the sensitivity power analysis available in G*Power 3 [63], with a beta value equal to 1 − alfa = 0.95 [64].

## 3. Results

The study cohort comprised 232 pregnant women. The age range was from 19 to 45 years (M = 29.18; SD = 4.41). The majority of women live in the city (n = 154; 67.0%), while the rest live in the village (n = 76; 33.0%). Most of women have a higher education (n = 146; 63.5%), the second largest group are women with a secondary education (n = 73; 31.7%), and only 11 women have a primary or vocational education (n= 11; 4.8%). More than half of the women are in their first pregnancy (n = 124; 53.9%). The second largest group are women who are in their second pregnancy (n = 64; 27.8%), 10% of women are in their third pregnancy (n = 23; 10%), while only 6.5% are in their fourth (n= 15; 6.5%), and only four women are in their fifth or later (n = 4; 1.7%). The pregnancy stage of the studied women varied, from 5 to 38 weeks (M = 20.28, SD = 7.32). For the vast majority of women, this will be their first delivery (n = 145; 63.0%), for 25.7% of women, this will be their second delivery (n = 59; 25.7%), for 7.8% of women, their third (n = 18; 7.8%), for 2.6% of women, their fourth (n = 6; 2.6%), and for only 0.9%, this will be their fifth or subsequent delivery (n = 2; 0.9%). Most of the women had not previously experienced serious problems, such as miscarriage or premature birth. Such reproductive problems occurred in 25.8% of the study participants (n = 50; 25.8%). Most of the pregnant women who took part in the study had no complications with previous deliveries (n = 169; 89.4%). As many as 3/4 of the women studied did not suffer from chronic diseases (n = 172; 74.2%).

First, descriptive statistics were analyzed for all variables studied. The Shapiro–Wilk test showed that most of the variables were characterized by a distribution other than normal. Despite the differences shown, the analysis of skewness and kurtosis showed that all distributions did not show significant asymmetry [65]. Considering the small asymmetry of the tested distributions and the size of the research sample, it was decided to use parametric analyses to verify the hypotheses, due to their statistical power [66]. Data on descriptive statistics are presented in Table 1.

**Figure 1:**
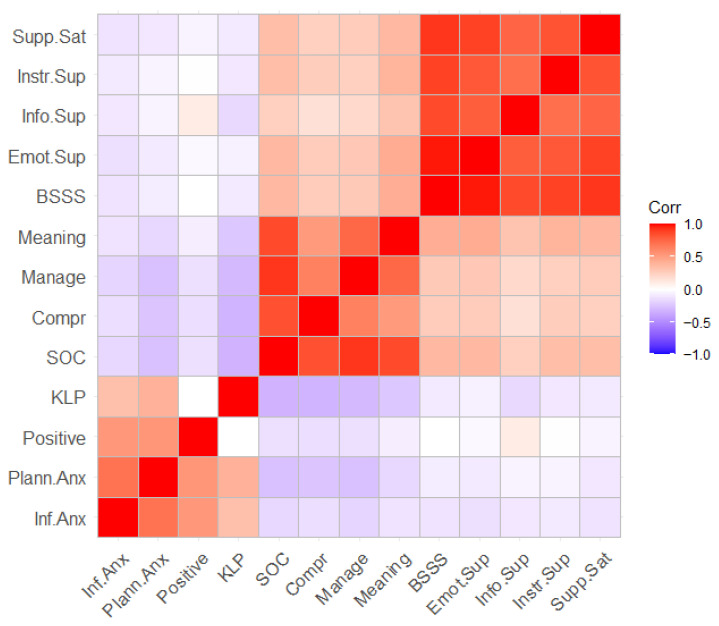
Graphical representation of correlation between birth anxiety, coherence, and social support. Note. (Supp sat)—satisfaction with support; (Instr. Sup)—instrumental support; (Info. Sup)—informational support; (Emot. Sup)—emotional support; (BSSS)—social support; (Meaning)—meaningfulness; (Manage)—manageability; (Comp)—comprehensibility; (SOC)—sense of coherence; (KLP)—fear of childbirth; (Positive)—Positive Appraisal; (Plann. Anx)—Preparedness Stress; (Inf. Anx)—Perinatal Infection Stress.

To check whether there is a relationship between the variables, Pearson r correlation analysis was used. The results are presented in Table 2 and in Figure 1, where a visualization of the full correlation matrix is presented.

The analysis showed that there are correlations between most of the variables. Prenatal stress (both related to infection and childbirth) is positively correlated with fear of childbirth and negatively correlated with the sense of coherence. Fear of childbirth negatively correlates with the sense of coherence and all of its components. It was also shown that there is a positive correlation between the sense of coherence and its components and all types of social support.

It was also checked whether there were any correlations between sociodemographic and obstetric variables and the analyzed psychological variables. The results are presented in Table 3.

Age has been shown to positively but weakly correlate with manageability. The number of deliveries negatively and weakly correlates with fear of childbirth and all types of social support. This means that the more times a woman has been pregnant, the less social support she receives, which is characterized by a lower sense of meaningfulness, and the less she is afraid of childbirth.

Fear of coronavirus infection correlates with stress related to infection and childbirth and with fear of childbirth. The more a woman fears of COVID-19, the more she is stressed and shows a higher level of fear of childbirth. Fear of hospitalization positively correlates with stress related to infection, childbirth, and fear of childbirth and negatively correlates with a sense of coherence, comprehensibility, and manageability. The importance of family childbirth correlates only with stress related to childbirth. The more importance a woman attaches to family childbirth, the higher the level of prenatal stress related to childbirth during the pandemic she shows.

An independent-samples *t* test was used to whether there were differences between the analyzed variables depending on the education of pregnant women. Respondents were divided into two groups based on their level of education (higher education vs. secondary or lower) to examine potential differences in psychological variables between women with different educational backgrounds, as educational attainment may influence access to information and coping resources. The data are presented in Table 4.

It has been shown that there are significant differences in the level of the sense of coherence and its components between women with higher education and women with secondary education. Women with higher education are characterized by a higher sense of coherence and all of its components. Significance sensitivity analysis using G*Power 3 [63] showed that for alfa = 0.05 and beta = 0.95, with a given number participants in each of the groups (Table 4), we needed a Cohen’s d value above 0.496 (d > 0.496); this criterion was not met. Some of the differences are close to the cutoff point, showing promise for the future research.

Then, we checked whether there are differences in the level of the analyzed variables between women who plan a family birth and those who do not plan such a birth. For this purpose, the parametric Student’s *t* test was used, and the results are presented in Table 5.

The analysis showed that there are significant differences between women planning a family birth and those who do not plan a family birth. Significant differences were found in the meaningfulness, as well as in emotional support and satisfaction with support. Women planning a family birth have a higher meaningfulness and receive more emotional support from their partner and are more satisfied with this support. Significance sensitivity analysis showed that for alfa = 0.05 and beta = 0.95, given the groups “Yes” (n = 152) and “No” (n = 77), we needed a Cohen’s d value above 0.507 (d > 0.507); this criterion was not met, and therefore, all of the results should be checked using greater sample sizes.

In the last step, it was decided to check whether there are differences between women who fear that their partner will not be able to be with them during childbirth and those who are not afraid of this. For this purpose, the parametric Student’s *t* test was used, and the results are presented in Table 6.

It has been shown that there are significant differences between women who fear the absence of a partner during childbirth and women who do not fear it. Women who fear the absence of a partner during childbirth experience higher levels of stress related to infection and childbirth, as well as higher levels of fear of childbirth. Significance sensitivity analysis showed that for alfa = 0.05 and beta = 0.95, given the groups “Yes” (n = 136) and “No” (n = 93), we needed a Cohen’s d value above 0.497 (d > 0.497); this criterion was met only by the Preparedness Stress scale, showing the true impact of partners’ presence during childbirth.

The last part of the analyses examined whether stress related to infection, stress related to childbirth, sense of coherence, and social support are explanatory variables of fear of childbirth in pregnant women. To achieve this goal, regression analysis was used, and the results are presented in Table 7.

There were no autocorrelations in the data (DW = 2.01, *p* = −0.926), and the variance inflation factor (VIF) for all of the variables was at a satisfying level. It was shown that stress related to childbirth is an explanatory variable for fear of childbirth for I-S (std β = 0.22, t = 12.98, *p* = 0.008), for P-S (std β = 0.37, t = 4.45, *p* < 0.001, and for Positive Appraisal (std β = −0.35, t = −5.13, *p* < 0.001). Data analysis also indicate that the sense of coherence is an explanatory variable for fear of childbirth (std β = −0.27, t = −4.27, *p* < 0.001). The regression model is significant (F = 19.48, *p* < 0.001, Adj. R^2^ = 0.29, f^2^ = 0.408). This model explains 30% of the variance in fear of childbirth and meets the significance sensitivity analysis criteria of f^2^(224) > 0.047.

## 4. Discussion

In the current study, we analyzed the relationship between pandemic stress, fear of childbirth, sense of coherence, and social support in pregnant women. We showed that there is a correlation between most of the analyzed variables.

Prenatal stress, both related to infection and the organization of childbirth, is correlated with fear of childbirth. The COVID-19 pandemic was associated with fear of infection and its complications, transmission of infection to the fetus, and hospitalization, but also quarantine and changes in the functioning of healthcare and restrictions in social and professional life. Many women had to give birth wearing a mask during the pandemic, some had to choose a different hospital than originally planned, others were isolated from the child after childbirth, and most had to give birth without a family member. All this information could lead to an increased fear of childbirth, which has also been confirmed in other studies [8,48]. Many studies conducted during the pandemic have shown that pregnant women experience higher levels of anxiety and are more stressed [67]. Given that the pandemic was both a threat to women’s health and the course of pregnancy, and also introduced restrictions on the functioning of maternity wards, it is not surprising that pandemic stress was associated with a higher level of fear of childbirth.

In the study, we also showed that women with higher levels of stress and fear of childbirth are more afraid of hospitalization during pregnancy. We also showed that stress related to preparing for childbirth is significantly correlated with the need for a family birth. This means that women who experience greater stress are afraid of both childbirth and hospitalization during pregnancy, need the support of a loved one more during childbirth, and are more afraid that they will be deprived of this support, which may also increase the fear of childbirth. Women who fear hospitalization are characterized by a higher fear of childbirth, higher pandemic stress, and a lower sense of coherence. Fear of hospitalization in the pregnancy pathology ward may be related to the presence of complications and a risk to the pregnancy. Studies indicate that women with a high-risk pregnancy are characterized by a higher level of fear of childbirth [68]. Fear of childbirth itself is associated with the fear of hospitalization, and the COVID-19 pandemic only intensifies the symptoms [67]. At the same time, anxiety can also occur as a trait, so a person will be afraid of the pandemic, hospitalization, complications, or difficult childbirth. Such people should be provided with special psychological care. At the same time, people with a low level of SOC may tend to perceive events as more threatening, and therefore show many concerns and fears [50].

In the study, we showed that women planning a family birth experience a significantly higher level of pandemic stress. This is most likely due to the fact that family births have been suspended due to the pandemic, especially with the increased number of illnesses [14]. Women who plan a family birth also rate their partner’s support higher. The presence of the child’s father during labor has a positive effect on the woman’s emotional state and on the course of the labor itself [5,14]. The literature has shown that the support of the partner, mother, other family members, and friends of a pregnant woman in the perinatal period is significant in reducing stress and anxiety [18,69]. We can assume that women planning a family birth may have a closer relationship with their partner and thus may receive more support from him. The current study also showed that women who fear not having a partner during labor are characterized by a higher fear of labor and a higher level of stress. However, other studies conducted on the Polish population showed that stress related to the organization of childbirth contributed to the development of negative feelings towards the partner [15].

Data analysis has shown that pandemic stress negatively correlates with the sense of coherence. Other studies have also shown a negative correlation between stress during the pandemic and the sense of coherence [50]. Many studies on various populations have also found that the sense of coherence is negatively correlated with the level of stress and fear during pandemic [70]. People with a high sense of coherence experience less anxiety [71]. On the other hand, people with a low level of SOC more often perceive situations as hopeless and see chaos in difficult situations [24]. According to Antonovsky, the sense of coherence reduces the likelihood of strong tension turning into stress, which was extremely important during the pandemic, and people with a strong SOC are less likely to assess situations as stressors, but more likely to treat them as harmless or indifferent, which allows them to avoid stress. Such people feel that they are able to cope with many situations [31,50]. Pregnant women with a high level of sense of coherence may have felt that regardless of the prohibitions and restrictions in the hospital, they will be able to cope during childbirth. The sense of coherence is an important resource at every stage of the stress transaction [72]; thanks to it, a person is able to activate the resources necessary for coping, which they can use effectively [24]. Pregnant women may have perceived the threat as smaller, and they may also have used the resources they possess to better prepare for childbirth. Women who were pregnant during the pandemic may have perceived both the pandemic itself and the restrictions associated with it as less threatening, and a high manageability may have made them feel that they would cope regardless of the circumstances that the pandemic brought.

The study also showed that the sense of coherence is negatively correlated with the level of fear of childbirth. This correlation has also been confirmed in many other studies [33,34,35,73,74]. Studies also show that the sense of coherence is correlated with lower levels of fear and anxiety among other groups of patients [75]. In the group of patients with cancer, SOC was found to be associated with lower emotional tension and lower levels of situational anxiety [76], and in people after leukemia, the sense of coherence was negatively correlated with the level of anxiety [77,78]. Lower fear and stress experienced by pregnant women with a higher level of sense of coherence and greater awareness of one’s own coping skills, associated with manageability, are associated with the fact that a woman experiences less fear and anxiety.

There was no relationship between fear of childbirth and social support, which is contrary to the results of other studies [79,80]. Most likely, partner support, in a situation of limited family births, is not associated with fear of childbirth. Only informational support was shown to be associated with the level of fear of childbirth. This is consistent with some studies indicating that informational support from midwives can reduce the level of fear of childbirth [81]. Small correlations were also shown between some types of social support and pandemic stress. Women who do not feel lonely during the pandemic and have the support of their relatives may perceive the pandemic situation as less threatening [8].

The study showed that the sense of coherence and all of its components positively correlate with social support and its subtypes. This means that the higher the level of sense of coherence in a pregnant woman, the higher she perceives the level of emotional, instrumental, and informational support she receives from her partner and the more satisfied she is with this support. This relationship has been confirmed in many other studies [82,83]. According to Antonovsky’s theory [24], both resources are interconnected. The sense of coherence allows one to use the resources, such as social support, in difficult and stressful situations in everyday life [83]. The study showed that the types of social support are most strongly associated with meaningfulness, the emotional and motivational component of the sense of coherence, expressing the belief in the meaning, value, and will to live. Women who feel that they are loved have a greater sense of meaning and will to live. Moreover, studies show that people with a high sense of coherence are usually also characterized by higher optimism and general satisfaction with life [84], which may also be associated with maintaining satisfactory social bonds.

The study also showed several significant correlations between sociodemographic variables and psychological variables. It was shown that age positively correlates with the sense of coherence. This correlation has been confirmed in several other studies [78]. According to Antonovsky’s [24] salutogenetic concept, the level of the sense of coherence is established after the age of thirty and is related to the resources possessed by an individual. This means that younger women who have not yet acquired resources such as education, material, or social status may be characterized by a lower level of it. Other studies also indicate that the level of personal resources increases with age [85]. Therefore, we have also shown that women with higher education are characterized by a higher sense of coherence. These data have been confirmed in the literature [86]. Education can be treated as an individual’s resource [24]. A better educated person has greater knowledge about the world and about the relationships between stimuli; therefore their sense of comprehensibility increases, and thus also their sense of coherence.

However, in our study, we showed that age negatively correlates with social support, which means that the older the person, the less social support they receive. Another study also showed that the level of social support decreases with age [87]. The study also showed that the number of pregnancies negatively correlates with social support. Perhaps women who are older and already have children do not receive as much support from their partners because they already have experience and no longer need help. Perhaps older women who already have children do not receive as much support, because it has to be distributed to other family members, including older children.

In the study, we also showed that the number of pregnancies negatively correlates with fear of childbirth, which means that women who already have experience are less afraid of childbirth itself. This is consistent with research results and theoretical concepts, according to which a higher fear of childbirth occurs in women who have not yet given birth [88]. The analysis also showed that the stage of pregnancy, expressed in weeks of pregnancy, correlates with the stress associated with infection. This means that women in more advanced pregnancy are more afraid of infection. This is most likely related to the fact that women in advanced pregnancy are preparing for childbirth, and COVID-19 infection in the perinatal period would affect the course of labor and possible isolation of the mother from the child [8]. In addition, women in advanced pregnancy are more susceptible to a more severe course of infection due to physiological changes.

In the next part of the analysis, it was decided to check whether pandemic stress, sense of coherence, and social support are explanatory variables for fear of childbirth. The study showed that stress related to childbirth, which is a component of pandemic stress, and sense of coherence are explanatory variables for fear of childbirth. Regression analysis is significant and explains 22% of the variance in the fear of childbirth variable.

During the pandemic, the provision of health services has changed significantly. In-person visits were limited and replaced by teleconsultations. The rules of care for pregnant women have also changed. Access to health services was difficult for a long time. It is not surprising that over 80% of women showed fear of childbirth during the COVID-19 pandemic [14]. Many women were unable to follow their previously prepared birth plan during the pandemic. The course of childbirth during the pandemic also raised concerns [89]. Studies suggest that a lack of control over decisions related to childbirth can be experienced as traumatic by women. In Poland, as in many other countries, family births have been restricted, despite the fact that support from a partner during labor is considered essential for the course of labor and for a woman’s well-being [5,49]. Pandemic stress related to the course of labor, lack of support from a loved one, and the possibility of being separated from the child is therefore a significant predictor of fear of childbirth, as has also been shown in other studies [8].

A significant predictor of fear of childbirth was also the sense of coherence, which is an important resource for coping with difficult situations. The sense of coherence is a resource that affects the perception of stressful situations. A person with a high level of sense of coherence perceives life events as less threatening and less stressful [50]. Women with a high level of sense of coherence may perceive childbirth as a situation they are able to cope with, and therefore, it is less threatening to them. Many studies have shown that people with a high level of sense of coherence are less likely to experience anxiety in difficult health situations [75,76,77,78]. They are convinced that they are able to cope even in the face of unexpected difficulties. There are studies that also indicate the role of sense of coherence as a significant predictor of fear of childbirth. Japanese studies presented a model that identified SOC as a direct predictor of fear of childbirth. These studies have shown that high SOC acts as a resilience factor that helps pregnant women cope with the stress of impending childbirth and reduces anxiety about childbirth [73]. The sense of coherence, as a resource important at every stage of the stress transaction, can reduce the tension that appears in connection with preparing for childbirth during the pandemic, and through manageability, it can make a woman feel that she is able to cope with childbirth regardless of the circumstances and pandemic restrictions. On the other hand, meaningfulness may make a woman feel that even if her delivery is more difficult or different from what she had planned due to the pandemic, it is worth the commitment and sacrifice for the child she is giving birth to.

Although our study provides interesting data on the importance of psychosocial resources for the functioning of pregnant women, it is not free from limitations. Its cross-sectional design does not allow for causal conclusions. Longitudinal studies would need to be conducted to fully verify the analyzed relationships. All variables were assessed using self-report questionnaires, which may introduce common method bias. Additionally, some potentially relevant confounders were not controlled for. Due to the fact that the study was conducted in Poland and that during the pandemic, in various countries, obstetric care was organized in various ways, it is difficult to transfer the obtained results to the situation in other countries. In subsequent studies, it would be worth analyzing the relationship between other personal resources and fear of childbirth, as well as other variables related to the functioning of pregnant women in the post-pandemic period. Despite these limitations, the study provides valuable insights into factors associated with fear of childbirth.

In summary, the study showed that pandemic stress is positively correlated with fear of childbirth. On the other hand, stress and anxiety are negatively associated with the sense of coherence and, to a small extent, with some types of social support. The study shows that obstetric variables related to the organization of childbirth are of significant importance for the mental functioning of women. The lack of control over such an important event as childbirth may contribute to a higher level of stress and the inability to use resources. The study showed that the sense of coherence and pandemic stress related to the organization of childbirth are explanatory variables for fear of childbirth. This is a very important result, which indicates how important the course of childbirth, appropriate care, and access to support for pregnant women are. In many Polish hospitals, perinatal care leaves many doubts, and women experience many difficult situations during childbirth, which may later affect their mental functioning. Therefore, one of the practical conclusions from this study is to allow women to give birth in a favorable atmosphere and in the company of a close person, if they need it. This may be of significant importance for reducing fear of childbirth.

## 5. Conclusions

The conducted study shows that the stress associated with organizing childbirth during the pandemic may contribute to increased fear of childbirth. Restrictions related to the functioning of maternity wards, characteristic of the pandemic, may be repeated during periods of increased incidence of seasonal infections, e.g., influenza. Therefore, taking into account the well-being of the mother and child and the importance of family support, it is worth allowing women to give birth in the presence of a companion during this time. In our study, we showed that the sense of coherence turned out to be a more important resource for the mental functioning of pregnant women than social support. These data confirm the assumptions of Antonovsky, who claimed that SOC is the most important resource for maintaining health, including mental health. A high level of the sense of coherence, which can reduce fear of childbirth, is a resource that protects pregnant women from experiencing negative emotions. The results of the current research can be used to develop assistance programs aimed at reducing fear of childbirth. They can also be used to work with women experiencing tokophobia. The results may be helpful for healthcare workers who provide support to women preparing for childbirth or hospitalized in pregnancy pathology wards.

The findings of this study underscore the important role of psychosocial resources, such as sense of coherence and social support, in shaping pregnant women’s psychological responses to stress and fear of childbirth, particularly during crises like the COVID-19 pandemic. Academically, these results highlight the need for further research into the mechanisms through which these resources operate and interventions designed to enhance them. Clinically, healthcare providers should assess stress levels, fear of childbirth, and available resources among pregnant women and consider strategies to strengthen a sense of coherence and social support, which may reduce fear and improve maternal mental health outcomes. From a policy perspective, it is important to maintain and promote social support during public health crises, for example, by facilitating partner presence during childbirth and ensuring access to psychosocial resources, thereby balancing infection control measures with the psychological well-being of expectant mothers.

## Figures and Tables

**Table 1:** Descriptive statistics of the studied variables with the Shapiro–Wilk test.

Variable	N	M	Min	Max	SD	SKE	K	W	*p*
Perinatal Infection Stress	230	12.1	5.26	5	24	0.22	−0.92	0.94	<0.001
Preparedness Stress	230	18.7	7.18	7	35	0.13	−0.88	0.97	<0.001
Positive Appraisal	230	6.21	3.02	3	15	0.63	−0.55	0.89	<0.001
Fear of childbirth	230	23.41	4.49	10	35	−0.16	−0.24	0.99	0.136
Sense of coherence	230	125.27	22.49	31	180	−0.31	0.76	0.99	0.03
Comprehensibility	230	42.94	8.96	11	66	−0.13	0.19	0.99	0.317
Manageability	230	43.53	9.11	11	67	−0.31	0.45	0.99	0.068
Meaningfulness	230	38.8	7.73	9	56	−0.35	0.21	0.99	0.03
Social Support	230	51.16	10.27	15	60	−1.45	1.4	0.81	<0.001
Emotional Support	230	30.84	6.2	9	36	−1.48	1.49	0.8	<0.001
Instrumental Support	230	10.23	2.18	3	12	−1.33	1.18	0.8	<0.001
Informational support	230	6.57	1.76	2	8	−1.08	0.09	0.79	<0.001
Satisfaction with support	230	3.52	0.8	1	4	−1.67	2.03	0.64	<0.001

**Table 2:** Relationship between stress (related to infection and childbirth), fear of childbirth, sense of coherence and its components, and social support (and its different types) in pregnant women.

Trait	I-S	P-S	POS	FOC	SOC	COM	MAN	MEAN	SS	EMO	INST	INF
I-S	---											
P-S	0.7 ***	---										
POS	0.53 ***	0.54 ***	---									
FOC	0.33 ***	0.4 ***	0	---								
SOC	−0.17	−0.27 ***	−0.13	−0.33 ***	---							
COM	−0.14	−0.25 **	−0.14	−0.32 ***	0.84 ***	---						
MAN	−0.18	−0.27 ***	−0.13	−0.3 ***	0.92 ***	0.64 ***	---					
MEAN	−0.12	−0.17	−0.07	−0.24 **	0.86 ***	0.52 ***	0.75 ***	---				
SS	−0.12	−0.08	0	−0.09	0.37 ***	0.27 **	0.28 ***	0.42 ***	---			
EMO	−0.13	−0.09	−0.03	−0.06	0.37 ***	0.27 ***	0.29 ***	0.43 ***	0.98 ***	---		
INST	−0.09	−0.05	−0.01	−0.1	0.34 ***	0.26 **	0.25 **	0.39 ***	0.89 ***	0.81 ***	---	
INF	−0.1	−0.05	0.1	−0.16	0.25 **	0.16	0.2 *	0.31 ***	0.86 ***	0.79 ***	0.72 ***	---
SAT	−0.12	−0.1	−0.05	−0.09	0.34 ***	0.25 **	0.27 ***	0.37 ***	0.92 ***	0.89 ***	0.83 ***	0.76 ***

* *p* ≤ 0.0025; ** *p* ≤ 0.0005; *** *p* ≤ 0.00005 (values adjusted with Bonferroni *p*-value adjustment for Pearson’s r correlation SD = 228). Note. I-S—Perinatal Infection Stress. P-S—Preparedness Stress. POS—Positive Appraisal. FOC—fear of childbirth. SOC—sense of coherence. COM—comprehensibility. MAN—manageability. MEAN—meaningfulness. SS—social support. EMO—emotional support. INST—instrumental support. INF—informational support. SAT—satisfaction with support.

**Table 3:** Relationship between sociodemographic and obstetric variables and stress (related to infection and childbirth), fear of childbirth, sense of coherence and its components, and social support (and its different types) in pregnant women.

Trait	I-S	P-S	POS	FOC	SOC	COM	MAN	MEAN	SS	EMO	INST	INF
Age	0.07	−0.02	0.02	−0.1	0.17	0.11	0.2 *	0.13	−0.16	−0.18	−0.09	−0.13
Num. of preg.	−0.03	−0.09	0.02	−0.18	−0.05	0	0.03	−0.17	−0.21 *	−0.23 **	−0.15	−0.16
Week of preg.	0.13	0.09	0.06	−0.08	0	0	−0.01	0	−0.08	−0.06	−0.05	−0.12
Num. of births	−0.04	−0.09	0.02	−0.26 **	−0.04	0	0.03	−0.14	−0.27 ***	−0.29 ***	−0.17	−0.21 *
Fear of COVID	0.57 ***	0.34 ***	0.24 **	0.16	−0.04	−0.06	−0.02	−0.01	−0.12	−0.11	−0.1	−0.11
Fear of hosp.	0.46 ***	0.4 ***	0.2 *	0.29 ***	−0.22 *	−0.2 *	−0.23 *	−0.13	−0.04	−0.07	0.02	−0.02
Importance of fam. birth	0.06	0.24 **	0.04	0.07	−0.01	−0.04	−0.02	0.04	0.05	0.04	0.09	0.04

* *p* ≤ 0.0025; ** *p* ≤ 0.0005; *** *p* ≤ 0.00005 (values adjusted with Bonferroni *p*-value adjustment for Pearson’s r correlation SD = 228); Note. I-S—Perinatal Infection Stress. P-S—Preparedness Stress. POS—Positive Appraisal. FOC—fear of childbirth. SOC—sense of coherence. COM—comprehensibility. MAN—manageability. MEAN—meaningfulness. SS—social support. EMO—emotional support. INST—instrumental support. INF—informational support. SAT—satisfaction with support. Num. of preg.—number of pregnancies. Week of preg.—week of pregnancy. Num. Of births—number of previous births. Fear of hosp.—fear of hospitalization. Importance of fam. Birth—the importance of family birth.

**Table 4:** Analyzed psychological variables and education level.

	Higher Education		Secondary Education		t	*p*	df	Cohen’s d
	(n = 146)		(n = 84)					
	M	SD	M	SD				
Perinatal Infection Stress	12.38	5.07	11.62	5.57	1.05	0.294	228	0.14
Preparedness Stress	18.86	6.76	18.43	7.9	0.43	0.665	228	0.06
Positive Appraisal	6.09	2.94	6.43	3.17	−0.82	0.413	228	−0.11
Fear of childbirth	23.6	4.31	23.07	4.79	0.87	0.383	228	0.12
Sense of coherence	129.07	21.07	118.67	23.47	3.46	<0.001	228	0.47
Comprehensibility	44.18	8.55	40.77	9.28	2.82	0.005	228	0.39
Manageability	44.97	8.48	41.02	9.67	3.22	0.001	228	0.44
Meaningfulness	39.92	7.19	36.87	8.29	2.93	0.004	228	0.4
Social support	51.66	9.98	50.29	10.77	0.97	0.333	228	0.13
Emotional support	31.31	5.94	30.02	6.6	1.52	0.131	228	0.21
Instrumental support	10.34	2.12	10.05	2.29	0.99	0.325	228	0.14
Informational support	6.46	1.8	6.76	1.66	−1.24	0.218	228	−0.17
Satisfaction with support	3.55	0.77	3.46	0.86	0.76	0.448	228	0.1

**Table 5:** Analyzed variables and planned family birth.

	Planned Family Birth							
	Yes		No		t	*p*	df	Cohen’s d
	(n = 152)		(n = 77)					
	M	SD	M	SD				
Perinatal Infection Stress	12.14	5.14	12.03	5.55	−0.16	0.872	227	−0.02
Preparedness Stress	19.41	7.17	17.31	7.11	−2.1	0.037	227	−0.29
Positive Appraisal	6.11	2.92	6.42	3.24	0.72	0.475	227	0.1
Fear of childbirth	23.52	4.5	23.23	4.5	−0.46	0.646	227	−0.06
Sense of coherence	127.2	22.1	121.3	23	−1.88	0.061	227	−0.26
Comprehensibility	43.39	9.02	42.01	8.87	−1.1	0.272	227	−0.15
Manageability	44.26	8.7	42.01	9.8	−1.77	0.078	227	−0.25
Meaningfulness	39.54	7.42	37.27	8.18	−2.11	0.036	227	−0.3
Social support	52.04	9.6	49.32	11.38	−1.9	0.059	227	−0.27
Emotional support	31.46	5.69	29.55	6.98	−2.21	0.028	227	−0.31
Instrumental support	10.41	2.1	9.88	2.32	−1.74	0.082	227	−0.24
Informational support	6.57	1.75	6.54	1.78	−0.14	0.892	227	−0.02
Satisfaction with support	3.6	0.72	3.35	0.93	−2.23	0.027	227	−0.31

**Table 6:** Analyzed variables with fear of not having a partner during childbirth.

	Fear of Not Having a Partner During Childbirth							
	Yes		No		t	*p*	df	Cohen’s d
	(n = 136)		(n = 93)					
	M	SE	M	SD				
Perinatal Infection Stress	13.07	5.31	10.7	4.9	−3.42	<0.001	229	−0.46
Preparedness Stress	21.79	6.26	14.18	6.01	−9.18	<0.001	229	−1.24
Positive Appraisal	6.61	3	5.63	2.99	−2.42	0.535	229	−0.33
Fear of childbirth	24.25	4.44	22.23	4.32	−3.42	<0.001	229	−0.46
Sense of coherence	123.15	22.83	128.23	21.85	1.68	0.092	229	0.23
Comprehensibility	42.52	8.91	43.53	9.08	0.83	0.330	229	0.11
Manageability	42.58	9.16	44.86	8.95	1.87	0.079	229	0.25
Meaningfulness	38.05	7.68	39.84	7.75	1.72	0.090	229	0.23
Social support	51.47	9.83	50.63	10.96	−0.61	0.597	229	−0.08
Emotional support	31.03	5.93	30.51	6.62	−0.62	0.573	229	−0.08
Instrumental support	10.32	2.09	10.11	2.33	−0.73	0.472	229	−0.1
Informational support	6.58	1.71	6.53	1.83	−0.24	0.991	229	−0.03
Satisfaction with support	3.54	0.77	3.48	0.85	−0.49	0.660	228	−0.07

**Table 7:** Explanatory variables of fear of childbirth.

						95% Confidence Interval			Model Fit				
Predictor	Beta	SE	t	*p*	Std β	Lower	Upper	VIF	F	*p*	R^2^	Adj. R^2^	*f* ^2^
Intercept	25.29	1.95	12.98	<0.001					19.48 (5.224)	<0.001	0.30	0.29	0.408
Perinatal Infection Stress (I-S)	0.19	0.07	2.68	0.008	0.22	0.06	0.38	2.13					
Preparedness Stress (P-S)	0.23	0.05	4.45	<0.001	0.37	0.21	0.54	2.24					
Positive Appraisal	−0.52	0.1	−5.13	<0.001	−0.35	−0.49	−0.22	1.53					
Sense of coherence	−0.05	0.01	−4.27	<0.001	−0.27	−0.39	−0.14	1.24					
Social support	0.03	0.03	1.06	0.289	0.06	−0.05	0.18	1.18					

## Data Availability

The data can be made available from the corresponding author upon reasonable request.
